# On-demand robotic surgery for hysterectomies: A combination of the best of robotic and laparoscopic approach

**DOI:** 10.1177/17455057251325029

**Published:** 2025-03-27

**Authors:** Sara Imboden, Damaris Erhardt, Siegenthaler Franziska, Mueller Michael David

**Affiliations:** Department of Obstetrics and Gynecology, Inselspital, Bern University Women’s Hospital, University of Bern, Bern, Switzerland

**Keywords:** Dexter, robot-assisted hysterectomy, flexibility, on-demand robot, gynecology

## Abstract

**Background:**

The advantage of robot-assisted hysterectomy over standard laparoscopy remains controversial. Conventional robots offer limited flexibility to the surgeon. The novel, Dexter robotic system™, allows integration and easy switch between the laparoscopic and robotic interface.

**Objectives:**

This study’s objective is to evaluate the feasibility of using Dexter for robot-assisted laparoscopic hysterectomies by analyzing surgical data and intra- and postoperative complication rates.

**Design:**

Retrospective study.

**Methods:**

Women who underwent surgery using the Dexter robotic system at a University Hospital in Switzerland from March to December 2022 were included in this study. A comprehensive database containing coded demographic and clinical outcome data for these patients was analyzed for surgical data and intra- and postoperative complications.

**Results:**

We collected and evaluated data from 24 patients who underwent Dexter robot-assisted hysterectomies for indications like endometrial cancer, endometriosis, and uterine fibroids. For all patients, a hysterectomy was accompanied by other procedures such as radical lymphadenectomy or deep infiltrating endometriosis surgery that was mostly performed by conventional laparoscopy. The mean operating time was 171.9 min, with an average estimated blood loss of 130.8 ml. The patients stayed at the hospital for an average of 4 days. Clavien–Dindo grade III postoperative complications were reported in four patients with three vaginal dome infections and one umbilical (camera arm) trocar hernia. Importantly, there were no intraoperative complications or conversion to laparotomy among the operated patients.

**Conclusion:**

We present the first retrospective study reporting the feasibility of Dexter in robot-assisted laparoscopic hysterectomies. All surgeries were performed successfully without technical failure or device-related adverse events. In contrast to the other robotic systems, Dexter offers an on-demand platform, allowing the surgeon to switch between laparoscopic and robotic interfaces as required. Further research is needed to validate its potential advantages and broader applicability.

## Introduction

Hysterectomy is one of the most common gynecological surgeries performed to resolve uterine fibroids, abnormal uterine bleeding, endometriosis, pelvic organ prolapse, and benign or malignant pathologies.^1^ Traditionally executed abdominally or vaginally, hysterectomy is now commonly performed using minimally invasive (MI) procedures due to their benefits such as less intraoperative blood loss, less pain, few postoperative complications, short stay at the hospital, and fast recovery.^2,3^

MI procedures are conducted either via classical laparoscopic hysterectomy (LH), which was first performed in 1988, or by using surgical robots that were first approved for gynecology in 2005.^4,5^ The advantage of one MI approach over the other remains controversial. Some studies demonstrate similar rates of complications, conversion to open surgery, and recovery time between LH and robotic-assisted hysterectomy (RAH), with RAH being more time-consuming and expensive.^6–8^ In contrast, a large multicenter and a 19-year-long, single-center retrospective study reported decreased intra- and postoperative complications with RAH.^9,10^

Despite the limited evidence showing the advantages of robotic surgery in gynecology, RAH is increasingly adopted in developed countries as it offers enhanced three-dimensional (3D) vision, precision of the wristed surgical instruments, easier training, and ergonomic benefits.^11,12^ With the growing use of surgical robots, significant development is ongoing to overcome their limitations like complicated docking processes, limited access of the patient by the clinician during surgery and high costs. The on-demand robot, Dexter, integrates with already available laparoscopic instruments like the trocars and camera, thus allowing an immediate switch between the robotic and laparoscopic interface by the surgeon who remains sterile throughout the procedure. The objective of this study was to investigate the feasibility of performing RAH using Dexter by analyzing surgical data and intra- and postoperative outcomes.

## Methods

### Ethics, subject selection, and study design

**Figure 1.**
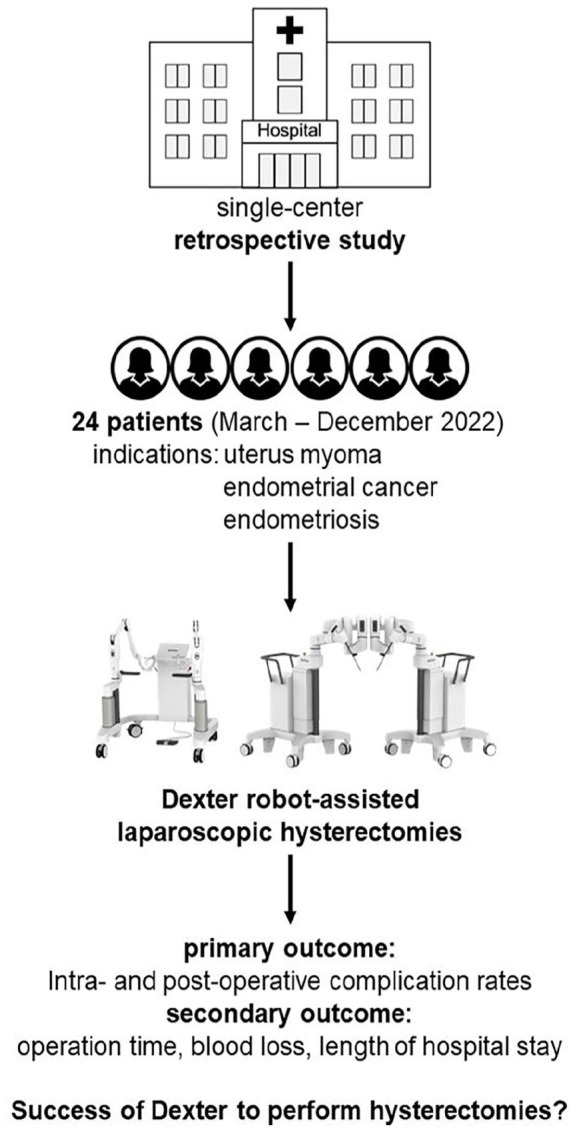
Patient flowchart.

The study was performed in accordance with the Swiss Human Research Act and Human Research Ordinance. Approval (number 2023-0216) from the institutional ethical committee was obtained for this single-center study. The STROBE checklist was used as a reporting guideline.^13^ In this retrospective observational study, we used clinical data collected during routine consultation and inpatient stays. All patients who underwent hysterectomy using Dexter robotic system at the University Hospital of Berne between March andDecember 2022 were included, with no additional exclusion criteria applied. The sample size of 24 was chosen due to the exploratory nature of this case series and logistical constraints. Results will be compared with existing literature. All surgeries were performed by a single surgeon with large laparoscopic experience, who had no previous experience with the Dexter system, but had experience with other robotic systems. Surgery with the Dexter robotic system was only possible once per week on a specific weekday, with additional schedule restrictions, which resulted in recruiting 24 patients with a hysterectomy on that specific day. Follow-up included consulting the clinical patient data at least for a duration of 1 year after surgery. Intra- and postoperative complication rates were defined as primary outcomes. Surgery-specific parameters such as operation time, estimated blood loss, and length of hospital stay were analyzed as secondary outcomes (Figure 1). Due to the small sample size, no group analyses were made. Permission to use the Dexter robotic system was granted by the copyright holder. This study has not previously been published but was presented at the Annual Congress of Swiss Gynecology as a video on June 29, 2023.

### Robot description

The Dexter robotic system™ (Distalmotion SA, Epalinges, Switzerland) is an open robotic platform that aims to enable a MI approach for complex surgeries.^14^ It was adopted as a new intervention at the study site. The robotic system consists of two patient carts—each equipped with a robotic arm—the endoscope cart with a robotic endoscope arm and the surgeon console. The endoscope arm is compatible with any 3D endoscope and the robotic arm for the camera is optional.

There are five robotic instruments available for robotic surgery with Dexter: a needle holder, a Johann grasper, a bipolar Maryland dissector, monopolar scissors and a monopolar hook that provide surgeons with natural dexterity and a greater range of motion. Furthermore, Dexter utilizes single-use sterile drapes and reusable accessories like incision pointers for docking, the handle grips of the surgeon console, and an emergency release tool. Dexter is fully integrated with the standard operating room equipment like radio frequency generator, insufflation device and 3D optics. Importantly, position of the trocars required for Dexter are similar to the laparoscopic surgery and is compatible with the usual 10 mm or larger diameter trocars. As in the laparoscopic setup, an assistant trocar can be positioned for additional support, such as for controlling retractors or suction devices. Unlike other robotic systems, Dexter allows surgeons to easily switch between LH and RAH modes, as it does not require complete undocking of the robotic system. Rather, the robotic arms include two modes, with the robotic arms being extended in the robotic mode, and the arms being folded into a compact formation, ensuring a seamless transition between the two modalities. When transitioning to laparoscopic mode, the surgeon can transition to the operating table without scrubbing, as the console is sterile.

### Surgery description

The basic laparoscopic surgery setup was used to perform RAH. The patients were operated on in lithotomy position. Electrical, insufflation devices, and screen positioning were similar to the ones used for laparoscopic surgery. The Dexter arms were draped and positioned on both sides of the patient. For personal preference, the endoscope was not held by the endoscope arm, but by an assistant. The surgeon’s console can be positioned anywhere in the room. In this study, the console was draped and positioned close to the patient. The surgeon remained sterile throughout the procedure, therefore allowing an easy transfer between the patient’s operation table and the surgeon’s console.

To begin the surgery, a manipulator and bladder catheter were placed, and the abdomen was accessed with a Veress needle. A uterine manipulator (HOHL or Rumi^®^ System by CooperSurgical Inc.) was used in 62.5% (15 out of 24) of the patients. Patients with high-risk endometrial cancer (EMCA) were operated without a uterine manipulator. After insufflation, the 10 mm trocars were placed at intraumbilical and parainguinal position and an assistant 5 mm trocar was placed suprapubic. When the robotic part of the surgery was started, the Dexter was docked as required. In this study two instrument arms on both sides of the patient were used. The goal of the surgery was to perform the hysterectomy using the Dexter system, while the remainder of the procedure was completed using conventional laparoscopy. Whenever laparoscopic access was preferred, Dexter arms were folded and positioned in the laparoscopic position, or the arms were distanced from the patient. The assistant handheld the camera and assisted the surgery over the suprasymphysal trocar. A detailed view of the surgery can be accessed in the Supplemental Video 1.

### Statistical analysis

A descriptive statistical analysis of the defined clinical outcomes was performed. The data are presented as mean value with the range and standard deviation. As relevant, the percentage of the patients affected was calculated. Normal distribution was tested using Shapiro–Wilk test. The two-tailed Student’s *t*-test was used to compare two datasets. One-way analysis of variance with Tukey’s post hoc analysis was used to compare three or more groups. A *p*-value of less than or equal to 0.05 was considered statistically significant.

## Results

**Figure 2.**
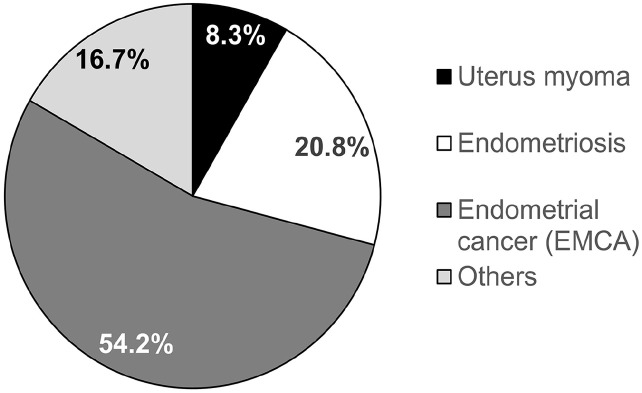
Distribution of surgery indications.

**Figure 3.**
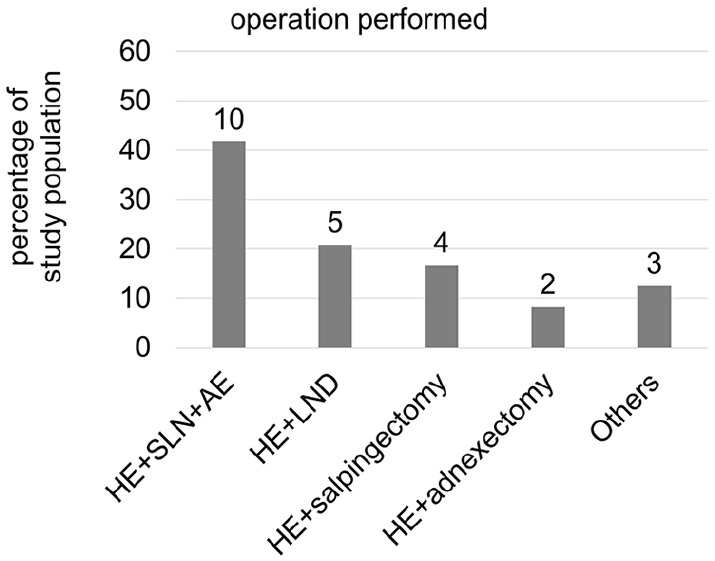
Distribution of surgeries performed.

We performed a retrospective study to evaluate the feasibility of the on-demand robotic system, Dexter, to perform RAH. A total of 24 women underwent Dexter-assisted laparoscopic hysterectomies. There was no missing data for any of the included variables. The average age of the patients at the time of surgery was 57.1 ± 14.5 years with a range of 35–85 years. One of the most common indications to perform hysterectomies was EMCA as diagnosed in 54.2% (13 out of 24) of the patients. About 20.8% (5 out of 24) of the women were diagnosed with endometriosis, 8.3% (2 out of 24) were operated for uterine myoma, and the remaining 16.7% (4 out of 24) showed other indications like endometrial polyp and borderline tumors of the ovary (Figure 2). The hysterectomy was accompanied with additional procedures in all patients. Along with RAH, 41.7% of the patients underwent simultaneous sentinel lymph node (SLN) and appendectomy, 20.8% underwent lymph node dissection (LND), 16.7% had salpingectomy, 8.3% underwent bilateral salpingo-oophorectomy, and the remaining 12.5% underwent other procedures like omentectomy or endometriosis removal (Figure 3).

None of the patients developed any intraoperative complications. RAH for all patients was completed successfully without technical failure and conversion to laparotomy. Out of 24 patients, 1 patient (4.2%) developed Clavien–Dindo scale grade I postoperative complication with the reported manifestation of genital herpes that was treated with Valacyclovir. Furthermore, 4 (16.7%) were diagnosed with Clavien–Dindo scale grade III postoperative complications (Table 1). One of these four patients was diagnosed with vaginal dome infection and secondary infected ascites that were treated with antibiotic therapy and ascites puncture. Two other patients with grade IIIb complications had vaginal dome infections, that required rehospitalization after 6–10 days of surgery for a diagnostic laparoscopy. The fourth patient with grade IIIb complication developed a trocar-site hernia, that needed to be treated surgically. The herniation occurred at the umbilical camera trocar site even though the fascia was closed with a Vicryl-0 stitch. The patient with the trocar hernia was treated for EMCA, while the vaginal dome infections were reported in two patients treated for endometriosis and one patient treated for EMCA. Notably, three of the four patients with Clavien–Dindo grade III complications had undergone additional surgical steps beyond the hysterectomy, including SLN, LND, and endometriosis resection.

**Table 1.** Patients with postoperative complications.

Grade I	1 (4.2)
Grade II	0
Grade IIIa	0
Grade IIIb	4 (16.7)
Grade IVa	0
Grade IV	0
Grade V	0

All values are shown as *n* (%), grade I–V: (Clavien–Dindo classification).

**Figure 4.**
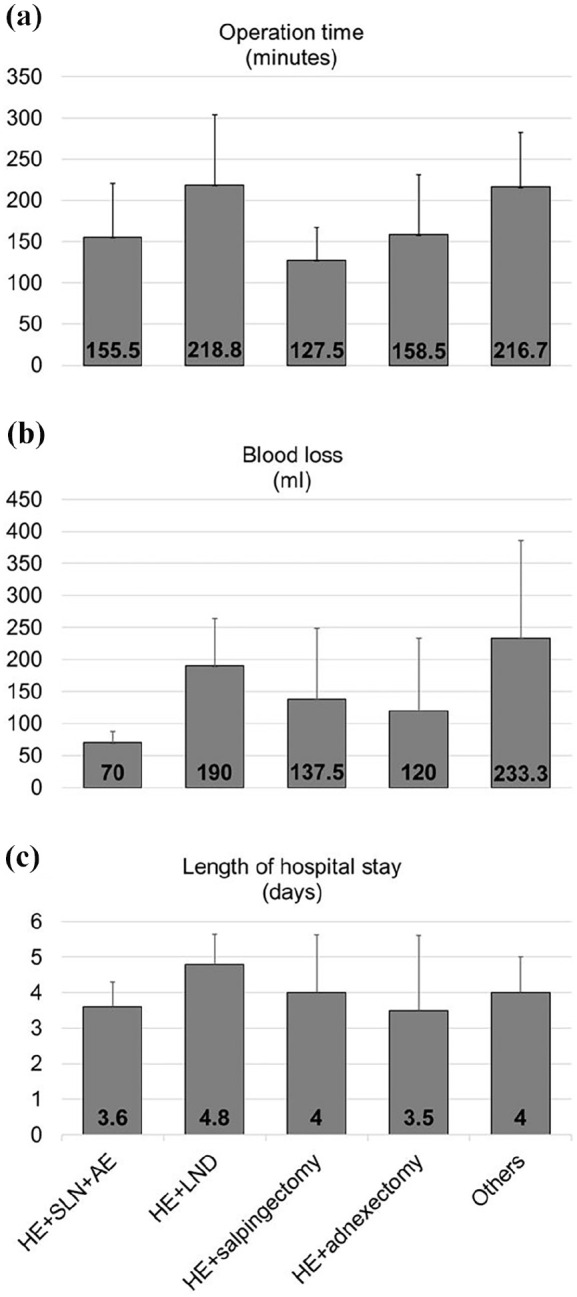
Outcomes stratified by surgery performed (a) Operation time (minutes), (b) Blood loss (milliliters), (c) Length of hospital stay (days).

**Figure 5.**
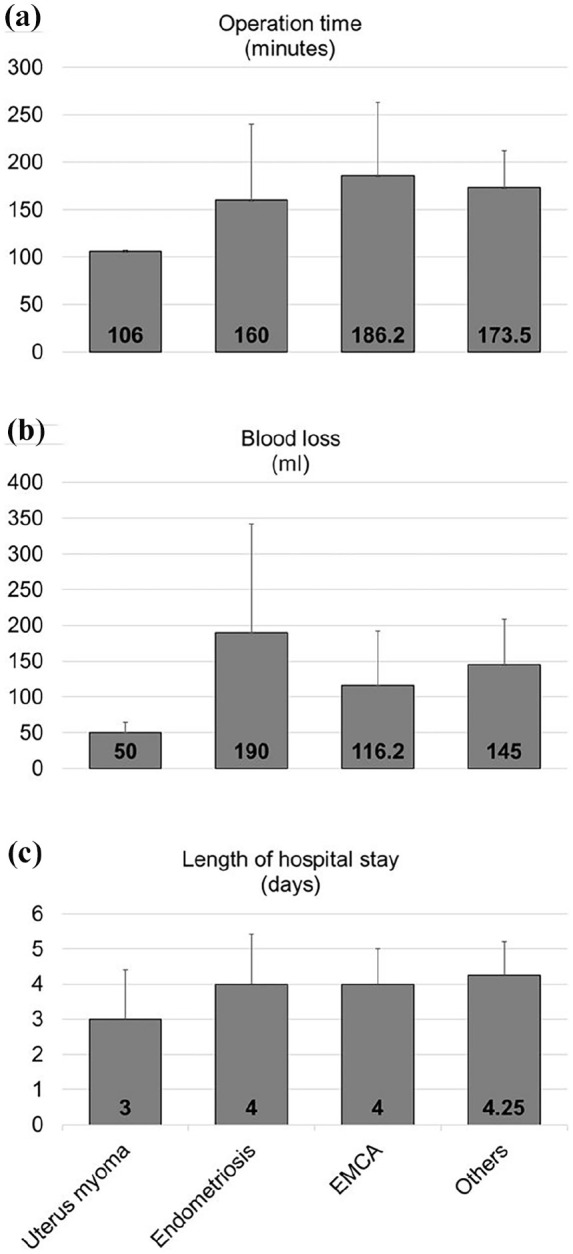
Outcomes stratified by surgery indication (a) Operation time (minutes), (b) Blood loss (milliliters), (c) Length of hospital stay (days).

The operation time, blood loss, and length of hospital stay were assessed as secondary clinical outcomes. The total operation time varied from 75 to 320 min with an average of 171.9 ± 70.3 min. The estimated blood loss varied widely from 40 to 400 ml, with a mean blood loss of 130.8 ± 95.0 ml among all patients. The patients stayed at the hospital for 2–6 days, with a mean length of stay of 3.9 ± 1.1 days (Table 2). The secondary outcomes of the study were not impacted by the additional operations performed (Figure 4) but showed a slight trend toward the indications (Figure 5). Patients with uterus myoma showed a comparatively short operation time of 106 ± 1.4 min as compared to 160 ± 80.4, 186.2 ± 74.0, and 173.5 ± 38.8 min required to operate patients with endometriosis, EMCA, and other indications, respectively. Similarly, patients with uterine fibroids lost only 50 ± 14.1 ml blood in comparison to 190 ± 152, 116.2 ± 72.8, and 145 ± 64 ml blood lost from the patients with endometriosis, EMCA, and other indications, respectively. However, there was no major difference in the length of stay at the hospital between the patients with different indications. Total stay at the hospital ranged from 3 days for the patients with uterine fibroids to 4 days for patients operated for endometriosis and EMCA or 4.25 for patients with other indications (Figure 5). It is to be noted that due to the low sample size of the study groups after segregation, no statistical analysis could be performed on these data and further larger studies would validate these findings.

**Table 2.** Secondary clinical outcomes.

Operation time (min)	171.9 (75–320; 70.3)
Estimated blood loss (ml)	130.8 (40–400; 95.0)
Length of hospital stay (days)	3.9 (2–6; 1.1)

All values are shown as mean (min–max; standard deviation).

Dexter was used for the hysterectomy in all patients. The rest of the surgery was mostly performed in the classical laparoscopic setting. This was surgeon’s choice, depending on the setup and goal of the surgery. In all cases, there was more than one switch between the robot and the classical laparoscopic setting, never causing any problems switching between the systems.

Since the surgeon always remained sterile, and the robot could be integrated into the existing laparoscopic workflow using the same trocars and camera, the switch between the laparoscopy and robot could be performed within seconds.

## Discussion

We present the first study demonstrating feasibility to use the on-demand robot, Dexter, to perform RAH. The reported clinical outcomes in our study do not deviate significantly from the previous publications with RAH. One study reported a mean operation time of 150 ± 180 min in 62 patients who underwent hysterectomies for benign conditions using the da Vinci Si^R^ or the da Vinci Xi^R^ robot.^6^ Other studies on RAH reported a mean operating time of 212 ± 57^15^ and 221.6 ± 94 min for EMCA and 108 ± 40 min for benign cases,^16^ with RAH generally requiring longer operating times than LH.^17^ The average length of hospital stay in these studies ranged from 5 and 11 days^15^ for EMCA and 3 days for benign cases.^16^ Costs were consistently higher for RAH than for LH.^17^ Depending on the indication, the estimated blood loss varied between 68 and 370 ml in different studies.^15,18^ The rate of vaginal cuff infections in our study was 12.5%, which is higher than the rate reported in the highly cited systematic review by Uccella et al.,^19^ which found up to 7.5% for both benign and malignant indications for conventional LH. It is also higher than the reported rate of up to 5.2% in robotic hysterectomy for malignant indications. However, due to the small sample size and the lack of a comparison group, there is a risk for bias and definitive conclusions regarding the safety of the robotic on-demand system cannot be drawn.

Studies have shown that MI hysterectomies are associated with less blood loss, short hospital stay, few pre- and post-operative difficulties with an improved quality of life as compared to open surgery for early-stage cervical cancer.^20,21^ In terms of oncological outcome for EMCA, a systemic review and meta-analysis study with a large sample size of more than 39,000 EMCA patients showed no difference in the overall rate of operative deaths caused by either LH or RAH, suggesting no difference in their postoperative outcomes.^7^ Additional single center studies show no difference between laparoscopic and robotic hysterectomies in terms of operation time, estimated blood loss, hemoglobin changes, length of hospital stay, and postoperative pain scores for the patients with benign condition.^22,23^ However, in obese patients with a body mass index greater than 30 or 40 kg/m², RAH was associated with longer operating times but lower conversion rates and shorter hospital stays compared to LH.^24,25^

Recent studies have shown that owing to the lack of advancements in the traditional laparoscopic surgeries, surgeons can benefit from the ergonomic advantages offered by the robotic systems with fast learning curves.^12,26^ Since the first robotic surgery performed in early 2000s, several other surgical robots like Senhance, Revo-i, Versius, Hugo RAS™, Avatera, and Hinotori have been increasingly developed and being used across the globe for MI operations.^27^

Although the evidence cannot prove the superiority of RAH over LH; however, a possibility to use the best of both approaches together would provide an increased advantage. The open, on-demand robotic platform, Dexter, was developed to achieve this aim. Unlike other fully robotic platforms that limit access to the operational setup, Dexter always ensures complete access to the patient during the surgery. Dexter’s instruments and compact size are designed to provide sufficient space for the surgeons and nurses to access the patient. Furthermore, the surgeon always remains sterile while working on a sterile console. The surgeon controls the camera navigation from the console with a possibility to switch it to manual operation as required. In contrast to other surgical robots, Dexter permits the same trocar position as required for the classical laparoscopic operation. The robot arms can be folded without the need for undocking thus making it possible to switch between the robotic to laparoscopic and vice versa within seconds. Such a flexible setup is potentially helpful for complex surgeries as it combines the robotic precision and dexterity while providing articulated instruments and full endoscopic control of the camera along with the standard operating room instruments available at any hospital equipped to perform conventional laparoscopy. Whether this application is beneficial remains to be analyzed and ultimately depends on the surgeon’s preference. In this study, the simpler part (hysterectomy) was often performed with the Dexter system, while the more complex procedures (endometriosis surgery, lymphadenectomy) were done laparoscopically. This approach was chosen due to the novelty of the Dexter system. During its implementation phase, we prioritized safety and performed only the simpler parts of the surgeries with the robotic system. However we chose not to limit patient selection excluding more complex indications, since we have the possibly to change back and forth between methods. Whether this flexibility is unnecessary or advantageous will likely remain a matter of debate among surgeons. Apart from its use in gynecology, other recent publications have already shown the success of Dexter to perform colorectal surgeries^28^ and prostatectomies.^29,30^

### Limitation

The primary limitation of this study is its preliminary nature, as reflected in the small sample size and the absence of a prior power analysis. Consequently, no comparison group was included, and the study lacked the statistical power to identify rare adverse events or draw definitive conclusions about specific types of surgeries or surgical indications.

## Conclusions

With this preliminary study on the application of Dexter in gynecology, we provide initial evidence suggesting the feasibility of performing hysterectomies with this novel on-demand system. One of the possible advantages offered by this open system includes an easy and on-demand switch between laparoscopic and robotic interface during specific steps as decided by the surgeon, possibly combining the best of robotic and laparoscopic approach. However, due to the study’s small sample size and lack of comparison group, the conclusions that can be drawn are limited. Further studies, involving larger sample sizes, comparison groups, prospective design, and multicentric recruitment, are needed to validate the advantages of this novel system.

## Supplemental Material

sj-pdf-1-whe-10.1177_17455057251325029 – Supplemental material for On-demand robotic surgery for hysterectomies: A combination of the best of robotic and laparoscopic approach Supplemental material, sj-pdf-1-whe-10.1177_17455057251325029 for On-demand robotic surgery for hysterectomies: A combination of the best of robotic and laparoscopic approach by Sara Imboden, Damaris Erhardt, Siegenthaler Franziska and Mueller Michael David in Women’s Health

## Declarations

**Ethics approval and consent to participate:** This research was ethically approved by the Swiss Association of Research Ethics Committees under reference number 2023-0216, on January 17, 2024. Written informed consent was obtained from all participants.

**Consent for publication:** Participants provided written consent for publication.

**Availability of data and materials:** The deidentified databases used in this project are available on request.

**Permission for using the Dexter robotic system:** Permission to use the Dexter robotic system was obtained from the copyright holder.
