# Supplementary material for: On-demand robotic surgery for hysterectomies: A combination of the best of robotic and laparoscopic approach
Source: Womens Health (Lond). 2025 Mar 27;21:17455057251325029. doi: 10.1177/17455057251325029 (PMC11952027; doi:10.1177/17455057251325029)
Supplement: sj-pdf-1-whe-10.1177_17455057251325029 – Supplemental material for On-demand robotic surgery for hysterectomies: A combination of the best of robotic and laparoscopic approach [file sj-pdf-1-whe-10.1177_17455057251325029.pdf]

STROBE Statement—checklist of items that should be included in reports of observational studies

	Item No	Recommendation	
**Title and abstract**	1	(*a*) Indicate the study's design with a commonly used term in the title or the abstract	Page 2, line 26
		(*b*) Provide in the abstract an informative and balanced summary of what was done and what was found	Page 1-2, lines 15-48
**Introduction**			
Background/rationale	2	Explain the scientific background and rationale for the investigation being reported	Page 3-4, lines 51-74
Objectives	3	State specific objectives, including any prespecified hypotheses	Page 4, lines 74-75
**Methods**			
Study design	4	Present key elements of study design early in the paper	Page 4-6, lines 76-148
Setting	5	Describe the setting, locations, and relevant dates, including periods of recruitment, exposure, follow-up, and data collection	Page 4, lines 82-95
Participants	6	(*a*) *Cohort study*—Give the eligibility criteria, and the sources and methods of selection of participants. Describe methods of follow-up<br>*Case-control study*—Give the eligibility criteria, and the sources and methods of case ascertainment and control selection. Give the rationale for the choice of cases and controls<br>*Cross-sectional study*—Give the eligibility criteria, and the sources and methods of selection of participants	Page 4, lines 82-84, 90-92
		(*b*) *Cohort study*—For matched studies, give matching criteria and number of exposed and unexposed<br>*Case-control study*—For matched studies, give matching criteria and the number of controls per case	Not applicable
Variables	7	Clearly define all outcomes, exposures, predictors, potential confounders, and effect modifiers. Give diagnostic criteria, if applicable	Page 4, lines 91-94
Data sources/ measurement	8*	For each variable of interest, give sources of data and details of methods of assessment (measurement). Describe comparability of assessment methods if there is more than one group	Page 2, lines 90-94
Bias	9	Describe any efforts to address potential sources of bias	Mentioning risk of bias in limitations, page 11-12, lines 275-278
Study size	10	Explain how the study size was arrived at	Page 4, lines 82-85, 88-90
Quantitative variables	11	Explain how quantitative variables were handled in the analyses. If applicable, describe which groupings were chosen and why	Page 4, lines 90-94
Statistical methods	12	(*a*) Describe all statistical methods, including those used to control for confounding	Page 6, lines 142-148
		(*b*) Describe any methods used to examine subgroups and interactions	Not applicable
		(*c*) Explain how missing data were addressed	Page 4, line 94
		(*d*) *Cohort study*—If applicable, explain how loss to follow-up was addressed<br>*Case-control study*—If applicable, explain how matching of cases and controls was addressed<br>*Cross-sectional study*—If applicable, describe analytical methods taking account of sampling strategy	Not applicable, there was no loss to follow-up
		(*e*) Describe any sensitivity analyses	Not applicable, no sensitivity analyses were performed

6 (a) There was no comparative group. However, we provided the description of the eligibility criteria for the single cohort recruited.

Continued on next page

Section	Item	Recommendation	Reported on
**Results**			
Participants	13*	(a) Report numbers of individuals at each stage of study—eg numbers potentially eligible, examined for eligibility, confirmed eligible, included in the study, completing follow-up, and analysed	Page 7 lines 151-152
		(b) Give reasons for non-participation at each stage	Not applicable
		(c) Consider use of a flow diagram	Not applicable
Descriptive data	14*	(a) Give characteristics of study participants (eg demographic, clinical, social) and information on exposures and potential confounders	Page 7, lines 152-166
		(b) Indicate number of participants with missing data for each variable of interest	Not applicable
		(c) *Cohort study*—Summarise follow-up time (eg, average and total amount)	Page 4, lines 90-91
Outcome data	15*	*Cohort study*—Report numbers of outcome events or summary measures over time	Page 7-8, lines 167-199
		Case-control study—Report numbers in each exposure category, or summary measures of exposure	
		Cross-sectional study—Report numbers of outcome events or summary measures	
Main results	16	(*a*) Give unadjusted estimates and, if applicable, confounder-adjusted estimates and their precision (eg, 95% confidence interval). Make clear which confounders were adjusted for and why they were included	Page 8, lines 184-197
		(*b*) Report category boundaries when continuous variables were categorized	Page 8 lines 184-197
		(*c*) If relevant, consider translating estimates of relative risk into absolute risk for a meaningful time period	Not applicable
Other analyses	17	Report other analyses done—eg analyses of subgroups and interactions, and sensitivity analyses	Not applicable
**Discussion**			
Key results	18	Summarise key results with reference to study objectives	Page 9-10, lines 215-217, 224-228
Limitations	19	Discuss limitations of the study, taking into account sources of potential bias or imprecision. Discuss both direction and magnitude of any potential bias	Page 11-12, lines 274-278
Interpretation	20	Give a cautious overall interpretation of results considering objectives, limitations, multiplicity of analyses, results from similar studies, and other relevant evidence	Page 12, lines 280-282
Generalisability	21	Discuss the generalisability (external validity) of the study results	Page 9-10, lines 217-139
**Other information**			
Funding	22	Give the source of funding and the role of the funders for the present study and, if applicable, for the original study on which the present article is based	Page 13, lines 304-305

*Give information separately for cases and controls in case-control studies and, if applicable, for exposed and unexposed groups in cohort and cross-sectional studies.

Note: An Explanation and Elaboration article discusses each checklist item and gives methodological background and published examples of transparent reporting. The STROBE checklist is best used in conjunction with this article (freely available on the Web sites of PLoS Medicine at http://www.plosmedicine.org/, Annals of Internal Medicine at http://www.annals.org/, and Epidemiology at http://www.epidem.com/). Information on the STROBE Initiative is available at www.strobe-statement.org.
